# Dissemination of Clonally Related *Escherichia coli* Strains Expressing Extended-Spectrum β-Lactamase CTX-M-15

**DOI:** 10.3201/eid1402.070350

**Published:** 2008-02

**Authors:** Teresa M. Coque, Ângela Novais, Alessandra Carattoli, Laurent Poirel, Johann Pitout, Luísa Peixe, Fernando Baquero, Rafael Cantón, Patrice Nordmann

**Affiliations:** * Hospital Universitario Ramón y Cajal, Madrid, Spain; † Unidad de Resistencia a Antibióticos y Virulencia Bacteriana Asociada al Consejo Superior de Investigaciones Científicas, Madrid, Spain; ‡ El Consorcio de Investigación Biomédica en Red de Epidemiología y Salud Pública, Madrid, Spain; § Istituto Superiore di Sanità, Rome, Italy; ¶ Hospital Bicetre, Paris, France; # Calgary Laboratory Services, Calgary, Alberta, Canada; ** University of Calgary, Calgary, Alberta, Canada; †† Universidade do Porto, Porto, Portugal

**Keywords:** CTX-M-15, IncFII plasmids, *Escherichia coli*, phylogenetic group B2, phylogenetic group D, extended-spectrum β-lactamase, research

## Abstract

We analyzed 43 CTX-M-15–producing *Escherichia coli* isolates and 6 plasmids encoding the *bla*_CTX-M-15_ gene from Canada, India, Kuwait, France, Switzerland, Portugal, and Spain. Most isolates belonged to phylogroups B2 (50%) and D (25%). An EC-B2 strain of clonal complex sequence type (ST) 131 was detected in all countries; other B2 isolates corresponded to ST28, ST405, ST354, and ST695 from specific areas. EC-D strains were clonally unrelated but isolates from 3 countries belonged to ST405. All CTX-M-15 plasmids corresponded to IncFII group with overrepresentation of 3 *Hpa*I-digested plasmid DNA profiles (A, B and C; 85–120kb, similarity >70%). Plasmid A was detected in EC*-*B2 strains (ST131, ST354, or ST405), plasmid C was detected in B2 and D strains, and plasmid B was confined to worldwide-disseminated ST131. Most plasmids contained *bla*_OXA-1_, *aac(6′)-Ib-cr*, and *bla*_TEM-1_. Worldwide dissemination of CTX-M-15 seems to be determined by clonal complexes ST131 and ST405 and multidrug-resistant IncFII plasmids.

## Abstract 2

*E*. *coli* ST131 and ST405 and multidrug-resistant IncFII plasmids may determine spread of this lactamase.

Plasmid-mediated CTX-M type expanded-spectrum β-lactamases (ESBLs), which have been extensively reported for the past 10 years, are detected mostly in community-acquired pathogens and are associated mainly with *Escherichia coli*. These β-lactamases compromise the efficacy of all β-lactams, except carbapenems and cephamycins, and are associated with many non–β-lactam resistance markers because of their locations on plasmids. Therefore, they may constitute a real threat for treating community-acquired *E*. *coli*–mediated urinary tract infections (*1*,*2*).

Different variants of CTX-M ESBLs are grouped in 5 clusters, although their distribution varies greatly depending on the geographic area (www.lahey.org/studies/webt.htm). CTX-M-15, which was first detected in isolates from India in 2001 (*3*), is now recognized as the most widely distributed CTX-M enzyme. It is derived from CTX-M-3 by 1 amino acid substitution at position 240 (Asp-240 → Gly), which apparently confers an increased catalytic activity to ceftazidime (*4*). Clonal outbreaks of CTX-M-15–producing *Enterobacteriaceae* have been reported in France, Italy, Spain, Portugal, Austria, Norway, the United Kingdom, Tunisia, South Korea, and Canada, and *E*. *coli* is the most frequently involved species. Within *E*. *coli*, CTX-M-15–producing strains of the B2 phylogenetic group are commonly found and frequently harbor multidrug resistance and virulence determinants (*5*–*18*).

Plasmids encoding *bla*_CTX-M-15_ have been isolated from clinical isolates in France, Spain, Portugal, the United Kingdom, Canada, India, Pakistan, South Korea, Taiwan, the United Arabic Emirates, and Honduras (*5*–*8*,*10*,*11*,*15*,*19*,*20*). Plasmid characterization, which has only been accomplished for those plasmids from Canada, France, Spain, and the United Kingdom, classified most of them as members of incompatibility group FII (*5*,*7*,*8*,*17*,*19*).

Lack of detailed studies on isolates expressing particular CTX-Ms from different geographic areas has precluded identification of factors involved in recent and worldwide spread of specific CTX-M variants. In this article, through analysis of the population biology of CTX-M-15–producing isolates from 7 countries and characterization of their genetic elements, we provide a comprehensive picture of elements involved in international spread of a particularly widespread mechanism of antimicrobial drug resistance.

## Materials and Methods

### Bacterial Strains, Production of ESBL, and Susceptibility Testing

We studied 43 CTX-M-15–producing *E*. *coli* clinical isolates from France (n = 17), Kuwait (n = 9), Switzerland (n = 7), Canada (n = 4), Portugal (n = 3) and Spain (n = 3), and 6 CTX-M-15 plasmids from India (*3*), all obtained from 2000 through 2006. These strains and plasmids were considered representative of these areas because they either caused outbreaks or were the first isolates recovered in those countries (*3*,*11*,*16*,*19*,*21*–*23*). Samples were isolated from urine (n = 33/43, 77%), wounds (n = 4/43, 9.%), respiratory tract infections (n = 3/43, 7%) and other sites (1 from feces, 1 from an intravenous catheter, and 1 from blood) in hospitalized patients. ESBL production was confirmed by a standard double-disk synergy test, and *bla* genes were characterized by PCR and additional sequencing as described (*19*). Susceptibility patterns to 13 non–β-lactam antimicrobial drugs were determined by the standard disk diffusion method following published standards (*24*). Strains with intermediate susceptibility were considered resistant.

### Clonal Relationships

Clonal relationships were established by pulsed-field gel electrophoresis (PFGE) of *Xba*I-digested genomic DNA (New England Biolabs, Ipswich, MA, USA) as described (*25*). Assignment of *E*. *coli* phylogenetic groups was conducted by using a multiplex PCR assay described by Clermont et al. (*26*). All *E*. *coli* isolates belonging to phylogroups B2 and D were characterized by multilocus sequence typing (MLST) using the standard 7 housekeeping loci (www.mlst.net). All *fumC* sequences from *E*. *coli* isolates belonging to phylogroup D were analyzed for a C288T single nucleotide polymorphism. This polymorphism is specific for a globally disseminated *E*. *coli* strain arbitrarily designated as *E*. *coli* clonal group A (CgA) that is associated with community-acquired urinary tract infections (*27*,*28*).

### Transferability and Location of *bla*_CTX-M-15_

Transferability was tested by broth and filter mating assays using *E*. *coli* K12 strain BM21R (resistant to nalidixic acid and rifampin, positive for lactose fermentation, and free of plasmids) as recipient at a 1:2 donor: recipient ratio. Transconjugants were selected on Luria-Bertani agar plates containing cefotaxime (1 mg/L) and rifampin (100 mg/L) and incubated at 37°C for 24–48 h. Transformation was performed for a subset of isolates by using conditions reported (*3*). Chromosomal or plasmid location of *bla*_CTX-M-15_ genes was assessed by hybridization of I-*Ceu*I–digested genomic DNA with *bla*_CTX-M-15_ and 16S rDNA probes and electrophoresis (5–25 s for 23 h and 60–120 s for 10 h at 14°C and 6 V/cm^2^) (*25*). Transfer and hybridization were performed by using standard procedures. Labeling and detection were conducted by using enhanced chemiluminescence (Amersham Life Sciences, Uppsala, Sweden) following manufacturer’s instructions.

### Plasmid Characterization

Plasmid DNA was obtained by using different midiprep plasmid purification kits (QIAGEN, Hilden, Germany, and Marlingen Biosciences, Ijamsville, MD, USA). Plasmids were classified according to their incompatibility group by a PCR-based replicon-typing scheme (*29*). Determination of plasmid size and confirmation of replicon content was established for transconjugants (or wild-type strains in the absence of transfer) by hybridization of S1 nuclease–digested genomic DNA with probes specific for *bla*_CTX-M-15_ and for different F replicons (FII, FIA, FIB), which were obtained by PCR as described (*19*). Relationships among plasmids were determined by comparison of *Eco*RI and *Hpa*I digested DNA patterns and comparison of repFII sequences. Genescan software (Applied Biosystems, Foster City, CA, USA) was used for collection of gel images. Data of a subset of representative patterns were exported into Fingerprinting II Informatix version 3.0 software (Bio-Rad Laboratories, Hercules, CA, USA) for further interpretation. Cluster analysis was conducted by using the unweighted pair group method with arithmetic averages (optimization 0.5%, tolerance 1.00%).

Presence of genes previously associated with plasmids encoding CTX-M-15 as *bla*_OXA-1_, *bla*_TEM-1_, and *aac(6′)-Ib-cr* was screened by PCR by using primers *bla*_OXA-1_ (oxa1 FW: 5′-TTT TCT GTT GTT TGG GTT TT-3′ and oxa1 RV: 5′-TTT CTT GGC TTT TAT GCT TG-3′), *bla*_TEM-1_ (TEM-F: 5′-ATG AGT ATT CAA CAT TTC CG-3′ and TEM-R: 5′-CTG ACA GTT ACC AAT GCT TA-3′), and *aac(6′)-Ib-cr* (aac-cr-F: 5′-TTG CGA TGC TCT ATG AGT GG-3′ and aac-cr-R: 5′-GCG TGT TCG CTC GAA TGC C-3′) (*11*,*19*,*30*). Additional sequencing was necessary to identify the corresponding genes.

## Results

### Epidemiologic Background

Most CTX-M-15–producing *E*. *coli* isolates belonged to phylogroups B2 (50%) and D (25%), which are known to be associated with the hospital setting and extraintestinal pathogenic *E*. *coli*. Phylogroups A (18%) and B1 (7%), which are associated with animal or human commensal strains, were less frequently represented. All isolates of phylogroups B2, A, and D corresponded to subgroups B2_3_, A_1_, and D_1_, respectively, which are the most common ones within each phylogenetic group (*31*). The 43 clinical isolates were classified into 32 PFGE types (B2_3_, 13; D_1_, 10; A_1_, 6; and B1, 3). Among B2_3_ strains, 10 PFGE types (18 isolates from France, Canada, Spain, Portugal, Kuwait, and Switzerland) were possibly related according to criteria of Tenover et al. (*32*) (difference <6 bands, >80% similarity) and were assigned to the sequence type (ST) ST131. The 4 unrelated B2 strains were classified within ST695 (1 from France), ST28 (1 from Switzerland), ST354 (1 from Portugal and Spain) and ST405 (1 from Portugal). All isolates of phylogroup D_1_ were clonally unrelated by PFGE (difference >6 bands), although MLST studies indicated that 4 PFGE types (5 isolates) from Kuwait, Switzerland, and Spain corresponded to ST405. The *fumC* sequences of the remaining 6 *E*. *coli* D strains were highly diverse (alleles 4, 13, 26, 88, and 132). None of the strains had the C288T single nucleotide polymorphism specific for *E*. *coli* strain CgA (*28*). All 3 B1 isolates were found in France. Among B2 *E*. *coli* isolates, all but 4 were isolated from urine and all but 2 belonged to ST131. These strains correspond to 2 isolates recovered from wounds and identified as ST28 and ST354 and 2 ST131 isolates from respiratory and fecal samples, respectively.

CTX-M-15 clinical strains were considered resistant to different antimicrobial drugs: amoxicillin-clavulanate (98%), tobramycin (89%), kanamycin (87%), tetracycline (84%), gentamicin (82%), nalidixic acid (74%), streptomycin (68%), sulfonamides (61%), ciprofloxacin (61%), trimethoprim (58%), chloramphenicol (21%), nitrofurantoin (12%), and amikacin (11%). All CTX-M-15 transconjugants expressed resistance to aminoglycosides, tetracycline, or trimethoprim. All but 2 strains contained *bla*_OXA-1_ and *aac(6′)-Ib-cr*; 1 contained only *aac(6′)-Ib-cr*, and 1 contained *bla*_OXA-1_ and *aacA4*, which confers reduced susceptibility to amikacin and kanamycin.

### Location and Transferability of *bla*_CTX-M-15_

The *bla*_CTX-M-15_ gene was located on plasmids in all but 6 strains and was positively transferred by conjugation or transformation in 37% of the strains tested. In 8 clinical isolates corresponding to 7 PFGE types, the probe for *bla*_CTX-M-15_ hybridized in chromosomal bands (2 belonging to B2_3_ ST131, 2 to D_1_, 1 to D_1_ ST405, and 1 to A_1_). In 2 other strains, the *bla*_CTX-M-15_ probe hybridized both with plasmid and chromosomal bands (1 strain from D ST405 and 1 from phylogroup B1).

### Plasmids Encoding CTX-M-15

Plasmids positive for the *bla*_CTX-M-15_ gene showed variable sizes (85–160 kb), belonged to the narrow host range incompatibility group IncF, and had replicon FII alone or in association with the FIA or FIB replicons (Appendix Table). Many restriction fragment length polymorphism (RFLP) patterns were observed, with overrepresentation of 3 profiles corresponding to 3 plasmids arbitrarily designated as plasmid A (85 kb), plasmid B (120 kb), and plasmid C (85 kb). Plasmid A, which was isolated from B2 *E*. *coli* strains from 4 countries (India, France, Portugal, and Spain), was associated with different STs (ST131, ST354, or ST405). Plasmid C was also detected in clonally unrelated *E*. *coli* of phylogroups B2 and D from Switzerland, Canada and France. Plasmid B, which was only associated with *E*. *coli* ST131, was widely disseminated in all countries studied. Sequence analysis of the replicons showed 4 repFII types: repFII(1), which was identical to that of plasmids R100, NR1, or pC15–1a, and was the most represented and identified in 23 plasmids; repFII(2), which had 99%–100% homology with plasmid pRSB107 (GenBank accession no. AJ851089), was identified in 6 plasmids; and repFII(3) and repFII(4), which were detected in 2 and 7 plasmids, respectively, and showed >93% homology with repFII(1). All repFIA and repFIB sequences were 99% and 100% homologous, respectively, with that of pRSB107 (GenBank accession no. AJ851089).

Computer analysis of representative RFLP patterns and repFII sequences grouped CTX-M-15 plasmids within 3 major clusters with similarity >70%. Cluster I comprises most plasmids, including plasmids A and B, most containing repFII(1) and showing variable replicon content. Cluster II comprised only plasmid C derivatives showing slightly different repFII sequences, and cluster III included 2 plasmids carrying repFII(2), FIA, and FIB replicons (Figure).

**Figure:**
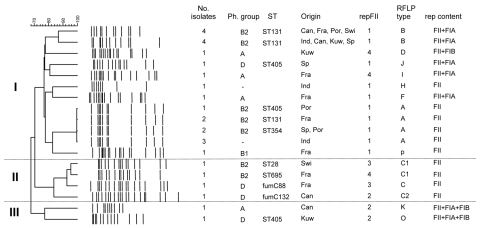
Computer analysis of a subset of representative *Hpa*I restriction profiles of IncF CTX-M-15 plasmids from *Escherichia coli* isolates in the Appendix Table. Cluster analysis was done by using Fingerprinting II in Informatix software version 3.0 (Bio-Rad Laboratories, Hercules, CA, USA) and applying the unweighted pair group method with arithmetic averages (optimization 0.5%, tolerance 1.00%). Ph, phylogenetic; ST, sequence type; RFLP, restriction fragment length polymorphism; Can, Canada; Fra, France; Por, Portugal; Swi, Switzerland; Ind, India; Kuw, Kuwait; Sp, Spain.

In the 8 strains with chromosomal location of *bla*_CTX-M-15_, repFII plasmids were identified but these plasmids were negative for the *bla*_CTX-M-15_ gene. Several strains that were also positive for additional plasmids and negative for the *bla*_CTX-M-15_ gene were assigned to different incompatibility groups or were untypeable by the PCR-based replicon typing scheme used.

## Discussion

Our study indicates that current worldwide spread of the *bla*_CTX-M-15_ gene is driven mainly by 2 epidemic *E. coli* strains belonging to phylogroups B2 (ST131) and D (ST405) and by its location on IncF plasmids harboring multiple antimicrobial drug–resistance determinants, including the recently described *aac(6′)-Ib-cr* gene. The presence of *bla*_CTX-M-15_ has previously been associated with *E*. *coli* strains of phylogroups B2 and D, and in some instances, with specific PFGE types (*9*–*12*,*16*). We detected an emerging and globally disseminated CTX-M-15 phylogroup B2 *E*. *coli* strain corresponding to the ST131 that was responsible for clonal outbreaks in Canada, France, Spain, and Portugal (*11*,*14*,*16*,*23*). Other CTX-M-15 B2 strains belong to clonal complexes ST695, ST405, ST354, or ST28, which have previously been detected in different geographic areas among isolates that do not express CTX-M-15 (Appendix Figure).

Globally disseminated *E*. *coli* strains associated with acute, uncomplicated, community-acquired cystitis and pyelonephritis, designated in community patients as clone CgA (ST69), have only been occasionally associated with CTX-M-15 production in Canada (*16*,*27*,*28*). Although the isolates in our study do not belong to clone CgA, they were isolated mainly from urine samples, and an association of ST131 *E*. *coli* isolates with urinary tract infections might be inferred. Although most CTX-M-15 isolates studied were recovered from hospitalized patients, these microorganisms are now widely spread in the community setting, including long-term care facilities in the countries from which isolates included in this study originated (*2*,*5*,*14*,*33*). Our study has increased knowledge of the number of epidemic *E*. *coli* clonal complexes causing urinary tract infections.

All plasmids carrying *bla*_CTX-M-15_ included in this study corresponded to incompatibility group F, and all had the FII replicon, which was assorted mainly in multireplicon plasmids with additional replicons of the FIA and FIB types. Association of the *bla*_CTX-M-15_ gene with IncFII replicons has been described in studies conducted in Canada, France, Spain, and the United Kingdom (*5*,*7*,*8*,*17*,*19*). Although we observed intercontinental dissemination of 3 major IncFII plasmid scaffolds (A, B, and C) carrying *bla*_CTX-M-15_, similarity >70% among all variants studied and presence of genes also found in pC15–1a, a CTX-M-15 plasmid (GenBank accession no. AY458016) that has a 28.4-kb multidrug resistance region containing *bla*_TEM-1_, *bla*_OXA-1_, the *aac(6′)-Ib-cr* gene (aminoglycoside 6′-N-acetyltransferase type Ib-cr variant responsible for reduced susceptibility to both aminoglycosides and certain fluoroquinolones), and genetic determinants coding for resistance to tetracycline and aminoglycosides (*5*,*30*), suggest a common origin or a common particular plasmid scaffold involved in the dissemination of CTX-M-15.

Because IncF plasmids are a heterogeneous and largely diffused family of plasmids in *E*. *coli*, they could acquire the *bla*_CTX-M-15_ gene. IncF plasmids negative for the *bla*_CTX-M-15_ gene in strains with this gene at a chromosomal location also suggest dynamic horizontal exchanges between the chromosome and resident plasmids. Extensive recombination events among IncF plasmids are frequent and may have contributed to their apparent high diversity (variable rep content, plasmid size, transferability, antimicrobial drug–resistance genes), driving their evolution and enabling them to persist in diverse *E*. *coli* populations (*34**,**35*). Such recombination events among plasmids of the same incompatibility group within the same cell occur frequently (*34**,**35*). This hypothesis is supported by the results of Lavollay et al. (*17*), who described mosaicism in a CTX-M-15 plasmid isolated in France that contained genes from 2 different IncFII plasmids, pC15–1a and pRSB107 (from IncFII plasmids first isolated from persons in Canada and activated sludge bacteria from a wastewater treatment plant in Germany, respectively) (*5*,*36*).

Spread and maintenance of conjugative plasmids across bacterial populations have been intensively studied from a theoretical point of view, but data from natural populations are scarce (*34*,*37*,*38*). Recovery of related plasmids from clonally unrelated B2 strains might reflect efficient transfer of these elements among different B2 *E*. *coli* populations. Sharing the same environment, successive immigrant B2 strains might sweep through the population, enabling plasmid hitchhiking at a high frequency in each selective sweep. However, we lack detailed information on the specificity and stability of different plasmid groups in specific hosts. An evolutionary convergent relationship among B2 genetic background and IncFII plasmids cannot be ruled out and should be studied because it might explain successful dissemination of CTX-M-15 plasmids within this *E*. *coli* lineage. In addition, our study is one of the few that have identified *bla*_ESBL_ genes in the chromosome, which might respond either to plasmid integration or transposition driven by IS*Ecp1* located upstream from the *bla*_CTX-M-15_ gene (*25*,*39*,*40*).

In conclusion, worldwide dissemination of *bla*_CTX-M-15_ is driven by B2 or D *E*. *coli* clones associated mainly with urinary tract infections or IncFII plasmids containing a multiple antimicrobial drug–resistance platform that contributes to spread of CTX-M-15. Further studies to test the stability/variability and fitness of particular plasmids among different bacterial hosts will be relevant in developing additional strategies to control dissemination of antimicrobial drug resistance.

## Supplementary Material

Appendix Table Epidemiologic data of CTX-M-15-producing Escherichia coli isolates from 7 countries*

Appendix Figure Geographic distribution of widely disseminated Escherichia coli clonal complexes associated with CTX-M-15. Data from strains lacking blaCTX-M-15 are from published studies (17,27,28; http://web.mpiib-berlin.mpg.de/mlst/dbs/Ecoli). E. coli clonal group A (CgA) has been identified as different sequence types (STs), most belonging to ST69 (27).
