# Supplementary material for: Dissemination of Clonally Related Escherichia coli Strains Expressing Extended-Spectrum β-Lactamase CTX-M-15
Source: Emerg Infect Dis. 2008 Feb;14(2):195–200. doi: 10.3201/eid1402.070350 (PMC2600198; doi:10.3201/eid1402.070350)
Supplement: Appendix Table — Epidemiologic data of CTX-M-15-producing Escherichia coli isolates from 7 countries* [file 07-0350_appT-s1.pdf]

Appendix Table. Epidemiologic data of CTX-M-15-producing *Escherichia coli* isolates from 7 countries*

									Plasmid features		
EC PhG	Location	Origin	Date	PFGE type	Isolate no.	MLST	Antimicrobial drug resistance pattern†	*bla*$_{CTX-M-15}$‡	Size, kb§	rep content¶	RFLP type#
$B2_3$	Portugal, Spain	Urine	2002–2003	C15–1	2	ST354	**Ac, Gm, Km,** Na, (Ni), **Sm, Su, Tb, Tp**	P (85)	**85**	**FII** (85)	A
$B2_3$	Portugal	Wound	2003	C15–2	1	ST405	**Ac, Gm, Km**, Na, Ni, **Sm, Su, Tb, Tp**	P (85)	**85**	**FII** (85)	A
$B2_3$	Paris, France, Portugal	Catheter (n = 1), urine (n = 1)	2001–2004	C15–3	2	ST131	Ac, Cp, **Gm, Km,** Na, Sm, Su, **Tc, Tb, Tp**	P (85)	**85**	**FII** (85)	A
–	India	–	2000	C15–4	1	–	**Ac, Gm, Km, Sm, Tb, Tc**	P (85)	**85**	**FII** (85)	A
–	India	–	2000	C15–5	1	–	**Ac, Gm, Km, Sm, Tb, Tc**	P (85)	**85**	**FII** (85)	A
–	India	–	2000	C15–6	1	–	**Ac, Gm, Km, Sm, Tb, Tc**	P (85)	**85**	**FII** (85)	A
–	India	–	2000	C15–7	1	–	**Ac, Gm, Km, Sm, Tb, Tc**	P (120)	**120**	**FII + FIA** (120)	B
$B2_3$	Kuwait	Urine	2004	C15–3a	1	ST131	Ac, Cp, Na, Te	P (120)	120	**FII + FIA** (120)	B
$B2_3$	Switzerland	Urine	2004	C15–3b	1	ST131	Ac, Cp, Gm, Km, Na, Tc, Tb	C	120	**FII + FIA** (120)	B
$B2_3$	Canada, Spain	Urine (n = 2)	2000–2002	C15–3c	2	ST131	Ac, Cp, Gm, Km, Na, Tc, Tb	P (120)	120	**FII + FIA** (120)	B
$B2_3$	Portugal, Canada, Paris, France	Urine (n = 2)	2001–2004	C15–3d	3	ST131	Ac, (Cp), **(Gm), (Km),** Na, **(Sm), (Tc), (Tb)**	P (120), n = 1; C, n = 2	120	**FII + FIA** (120)	B
$B2_3$	Paris and Reims, France	Urine (n = 3), feces (n = 1)	2003–2005	C15–3e	4	ST131	Ac, (Cp), **(Gm), (Km),** Na, **(Sm), (Tc), (Tb)**	C	120	**FII + FIA** (120)	ND
$B2_3$	Reims, France	Urine	2005	C15–3f	1	ST131	Ac, Cp, Na	P (150)	**150**	**FII + FIA + FIB** (150)	FE
$B2_3$	Kuwait	Urine	2004	C15–3g	1	ST131	Ac, Cp, Gm, Km, Na, Sm, Tc, Tb, Su, Tp	P (100)	100 + 150	**FII** + FIB (100)	ND
$B2_3$	Switzerland	Urine	2004	C15–3h	1	ST131	Ac, Cp, Gm, Km, Na, Su, Tc, Tb, Tp	P (160)	160	**FII + FIA** (160)	N
$B2_3$	Paris, France	Urine	2000	C15–8	1	ST695	Ac, Na, Sm, Su, **Tc,** Tp	P (85)	**85**	**FII** + FIB (85)	C1
$B2_3$	Switzerland	Wound	2004	C15–9	1	ST28	Ac	P (85)	**85** + 120	**FII** (85)	C1
D_1	Switzerland	Respiratory tract	2004	C15–10	1	*fumC* 13	Ac, Gm, Km, Tc, Tb,	P (110)	110	**FII** + FIB (110)	E
D_1	Paris, France	Urine	2000	C15–11	1	*fumC* 26	Ac, Ak, Cp, Cm, Gm, Km, Na, Sm, Su, **Tc,** Tb, **Tp**	P (100)	**100**	**FII** (100)	ND
D_1	Spain	Wound	2002	C15–12	1	ST405	Ac, Cm, Gm, Km, Na, Sm, Su, Tc, **Tb, Tp**	P (100)	**100** + 120	**FII + FIA** (100)	J
D_1	Canada	Urine	2000	C15–13	1	*fumC* 132	Ac, Cm, Cp, Gm, Km, Na, Sm, Su, Tb, Tp	P (85)	85 + 120	**FII** (85)	C2
D_1	Kuwait	Wound	2004	C15–14	1	*fumC* 4	Ac, Cp, Na, Sm, Su, Tc, Tp	C	120 + 100	**FII + FIA + FIB** (120)	ND
D_1	Switzerland	Urine	2004	C15–15	2	ST405	Ac, Cp, Gm, Km, Na, Sm, Su, Tc, Tb, Tp	C	85 + 120	**FII + FIA + FIB** (85)	ND

D_1	Kuwait	Respiratory tract	2004	C15–16	1	ST405	Ac, Cp, Gm, Km, Na, Sm, Su, Tc, Tb, Tp	P (150)	150	**FII + FIA + FIB** (150)	O
D_1	Kuwait	Urine	2004	C15–17	1	ST405	Ac, Ak, Cp, Gm, Km, Na, Sm, Su, Tc, Tb, Tp	P (160), C	160	**FII + FIA + FIB** (160)	ND
D_1	Switzerland	Urine	2004	C15–18	1	*fumC* 26	Ac, Cp, Gm, Km, Na, Su, Tc, Tb, Tp	C	**120**	**FII + FIA + FIB** (120)	L
D_1	Paris, France	Urine	2001	C15–19	1	*fumC* 88	Cm, Cp, Gm, Na, Su, Tc, Tb, Tp	P (85)	**85**	**FII** (85)	C
–	India	–	2000	C15–20	1	–	**Ac, Cm, Sm**	P (85)	**85**	**FII** (85)	G
–	India	–	2000	C15–21	1	–	**Ac, Gm, Km, Sm, Tc, Tb**	P (85)	**85**	**FII** (85)	H
A_1	Kuwait	Blood	2004	C15–22	1	–	Ac, Gm, Km, Sm, Tc, Tb	–	–	**FII**	FE
A_1	Paris, France	Urine	2001	C15–23	1	–	Ac, Ak, Cm, Cp, Gm, Km, Na, Ni, Sm, Su, Tc, Tb, Tp	C	100	**FII + FIA + FIB** (100)	F
A_1	Canada	Urine	2001	C15–24	1	–	Ac, Cp, Gm. Km, Na, Sm, Su, Tc, Tb	P (110)	110	**FII + FIA + FIB** (110)	K
A_1	Kuwait	Respiratory tract (n = 1), urine (n = 1)	2004	C15–25	2	–	Ac, (Ak), Cp, Gm, Km, Na, (Ni), (Sm), Su, Tc, Tb, Tp	P (120)	120	**FII + FIA** (120)	N
A_1	Reims, France	Urine	2005	C15–26	2	–	Ac, Cm, Cp, Gm, Km, Na, Su, Tb, Tp	P (120)	**120** + 100	**FII + FIA + FIB** (120)	ND
A_1	Kuwait	Urine	2004	C15–27	1	–	Ac, Cp, Km, Na, Sm, Su, Tc, Tb, Tp	P (85)	85 + 100	**FII + FIB** (85)	D
B_1	Paris, France	Urine	2003	C15–28	1	–	Ac, Km, Tc, Tb	P (85)	85	**FII** (85)	M
B_1	Paris, France	Urine	2003	C15–29	1	–	Ac, Km, Sm, Su, **Tc, Tb,** Tp	P (80)	**80**	**FII** (85)	P
B_1	Paris, France	Urine	2002	C15–30	1	–	Ac, Cm, Cp, Gm, Km, Na, Ni, Sm, Su, Tc, Tb, Tp	P (100), C	100 + 40	**FII + FIA** (100)	I

*EC PhG, *E. coli* phylogenetic group; PFGE, pulsed-field gel electrophoresis; MLST, multilocus sequence typing; RFLP, restriction fragment length polymorphism; ST, sequence type.
†Transferability of antimicrobial drug resistance to a given drug is indicated in **boldface**. Drugs in parentheses are not associated with resistance in all isolates. Ac, amoxicillin-clavulanic acid; Gm, gentamicin; Km, kanamycin; Na, nalidixic acid; Ni, nitrofurantoin; Sm, streptomycin; Su, sulfonamide; Tb, tobramycin; Tp, trimethoprim; Tc, tetracycline; Cp, ciprofloxacin; Ak, amikacin; Cm, chloramphenicol.
‡Chromosomal (C) or plasmid (P) location of the $bla_{CTX-M-15}$ gene was determined by hybridization of probes with I-*Ceu*I-digested genomic DNA. Size of the plasmid in kb is indicated in parentheses.
§Plasmid size and content were determined by hybridization of S1 nuclease–digested genomic DNA on transconjugants (or wild type if transfer failed). Successful plasmid transfer by conjugation or transformation is indicated in **boldface**.
¶Replicon content of plasmids with $bla_{CTX-M-15}$ genes was determined by PCR, hybridization, and sequencing of replicons. Replicons hybridizing in the same band as that of the $bla_{CTX-M-15}$ gene are indicated in **boldface**. Numbers in parentheses represent plasmid sizes in kb.
#ND, not done; FE, failed extraction.
