# Supplementary figures and images for: Dissemination of Clonally Related Escherichia coli Strains Expressing Extended-Spectrum β-Lactamase CTX-M-15
Source: Emerg Infect Dis. 2008 Feb;14(2):195–200. doi: 10.3201/eid1402.070350 (PMC2600198; doi:10.3201/eid1402.070350)

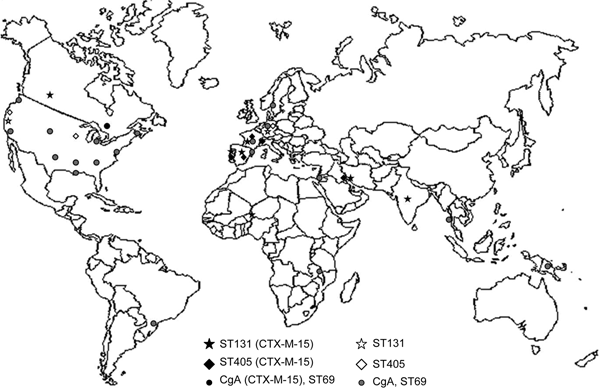

Supplement: Appendix Figure — Geographic distribution of widely disseminated Escherichia coli clonal complexes associated with CTX-M-15. Data from strains lacking blaCTX-M-15 are from published studies (17,27,28; http://web.mpiib-berlin.mpg.de/mlst/dbs/Ecoli). E. coli clonal group A (CgA) has been identified as different sequence types (STs), most belonging to ST69 (27). [file 07-0350_app-s2.gif]
